# Are prophylactic anti-reflux medications effective after esophageal atresia repair? Systematic review and meta-analysis

**DOI:** 10.1007/s00383-018-4242-4

**Published:** 2018-03-13

**Authors:** Hiromu Miyake, Yong Chen, Alison Hock, Shogo Seo, Yuhki Koike, Agostino Pierro

**Affiliations:** 1 Division of General and Thoracic Surgery, The Hospital for Sick Children, 1526-555 University Avenue, Toronto, ON M5G1X8 Canada; 2 Department of Pediatric Surgery, Shizuoka Children’s Hospital, 860 Urushiyama, Aoi-ku, Shizuoka, 4208660 Japan

**Keywords:** Esophageal atresia, Gastroesophageal reflux, Esophageal stricture, Anti-reflux medicine, Proton pump inhibitor, H_2_ blocker

## Abstract

**Purpose:**

Gastroesophageal reflux after surgical repair of esophageal atresia (EA) can be associated with complications, such as esophageal stricture. Recent guidelines recommend prophylactic anti-reflux medication (PARM) after EA repair. However, the effectiveness of PARM is still unclear. The aim of this study was to review evidence surrounding the use of PARM in children operated for EA.

**Methods:**

We performed a systematic review and meta-analysis. We searched Medline, EMBASE, and the Cochrane Databases from inception until the end of 2016 for comparative studies of PARM versus no PARM (control). Primary outcome was postoperative esophageal stricture. Quality of evidence was assessed using GRADE system.

**Results:**

We identified four observational studies that focused on esophageal stricture as an outcome. A total of 362 patients were included in meta-analysis. There was no significant difference in esophageal stricture rates between PARM and control (OR = 1.14; 95% CI = 0.61–2.13; *p* = 0.68; *I*^2^ = 38%). The quality of the evidence was very low, due to lack of precision as a consequence of small study sizes.

**Conclusions:**

Our results indicate that PARM does not reduce the incidence of esophageal stricture after EA repair. Future well-controlled prospective studies are needed to obtain higher quality evidence.

## Introduction

Esophageal atresia (EA) is a relatively rare congenital malformation that occurs in approximately 1 for every 2500–4500 births [1–3]. Owing to improvements in surgical and perioperative management, a survival rate of more than 90% has been achieved. However, complication rates still remain high. Post-anastomotic esophageal stricture is one of the most frequent complications after EA repair, with an incidences of approximately 40% [1–4]. Postoperative gastroesophageal reflux (GER) was known to be associated with esophageal stricture [4–6]. GER is also known to cause respiratory complications such as recurrent pneumonia, failure to thrive, respiratory distress and apparent life-threatening events [7]. Thus, treatment of GER is of critical importance to reduce complications after EA repair. Recent surveys have revealed that the majority of patients after EA repair are prescribed prophylactic anti-reflux medications (PARMs), such as proton pump inhibitor (PPI) or H_2_ blocker, even before GER symptoms develop [8–10]. However, there is a lack of evidence to justify the use of PARM in these patients. The aim of this report was to review the current evidence for the use of PARM in patients after EA repair.

## Methods

We followed the Cochrane handbook for systematic reviews of intervention and the preferred reporting item for systematic reviews and meta-analysis (PRISMA) statement for this systematic review and meta-analysis [11, 12]. We searched articles from January 1946 to December 2016 in the MEDLINE, EMBASE, and Cochrane Central Register of Controlled trials using combinations of the following terms: “esophageal atresia,” “gastroesophageal reflux,” “esophageal stricture,” and “anti-reflux medicine.” In addition, a manual search of the references of retrieved articles was performed. We planned to include all published observational studies and randomized controlled trials (RCTs). Postoperative esophageal stricture was the main outcome for this meta-analysis. We considered recurrent pneumonia as secondary outcome. We included all studies comparing outcomes in patients with PARM and without PARM (control) after EA repair. PARM was defined as administration of anti-reflux medication within a week of EA repair. We excluded studies that overlapped with later publication. Although we applied no language restriction, all articles included in this meta-analysis were published in English.

Two reviewers (HM and YC) independently screened all retrieved reports with a low threshold for selecting studies for full-text review. Full texts were then independently reviewed to identify included studies. In this step, we extracted the following data from each article: first author and year of publication, study design, country, years of study, sample size, type of EA, number of patients with anastomotic leak, number of patients with long gap EA, type of PARM, dose of PARM, duration of PARM, follow-up period, and outcomes. Disagreement regarding inclusion was resolved by a discussion between reviewers, reaching consensus at each stage of screening process.

We performed the meta-analysis using Review manager 5.3. We estimated statistical significance using a two-sided *p* value of 0.05. Effect sizes were calculated and presented as pooled odds ratio (OR) along with a 95% confidence interval (CI). Because heterogeneity among the studies was anticipated, a random-effects model was implemented using the Inverse Variance method.

The grading of recommendations and assessment, development and evaluation (GRADE) system was used to assess the quality of the evidence [13–20]. Quality of evidence was rated as high, moderate, low, and very low for each outcome. Observational studies start with a low quality of evidence. The quality of evidence was rated down in the presence of risk of bias, inconsistency, indirectness, imprecision, and publication bias. For assessment of risk of bias in observational studies, Newcastle–Ottawa Scale (NOS) was used [21]. Two of the authors (HM and YC) independently assessed risk of bias. The most important confounder was the presence of long gap [5]. Secondary important confounders were type of EA (Gross classification) [22], anastomotic leak, anastomotic tension, primary anastomosis, and birth weight. The confounders were identified by one investigator, who is also a pediatric surgeon (HM). Each confounder was analyzed between PARM and control group using GraphPad Prism 6 (GraphPad Software Inc., San Diego, CA, USA). Categorical data were analyzed using the Chi-squared and Fisher’s exact tests. *P* values of < 0.05 were considered significant. As there is no set cut-off score, we selected a score ≧ 7 as indication of low risk of bias. Inconsistency was determined according to heterogeneity. *I*^2^ statistics was used to determine heterogeneity. *I*^2^ value of 0–40, 30–60, 50–90, and 75–100% were considered as low, moderate, substantial, and considerable heterogeneity, respectively [11]. Imprecision was assessed using optimal information size (OIS), which was based on 20% relative risk reduction, 0.05 of αerror and 0.20 of βerror [23]. We planned to assess publication bias using funnel plots if ten or more studies were available. The quality of evidence was upgraded in the presence of large magnitude of effects, dose–response gradient, and plausible confounders. Large magnitude of effect was present if relative risk (RR) was greater than 2 or less than 0.5. We summarized the results of the meta-analyses and the assessment of quality of evidence for each outcome using GRADEpro GDT [24].

## Results

**Fig. 1:**
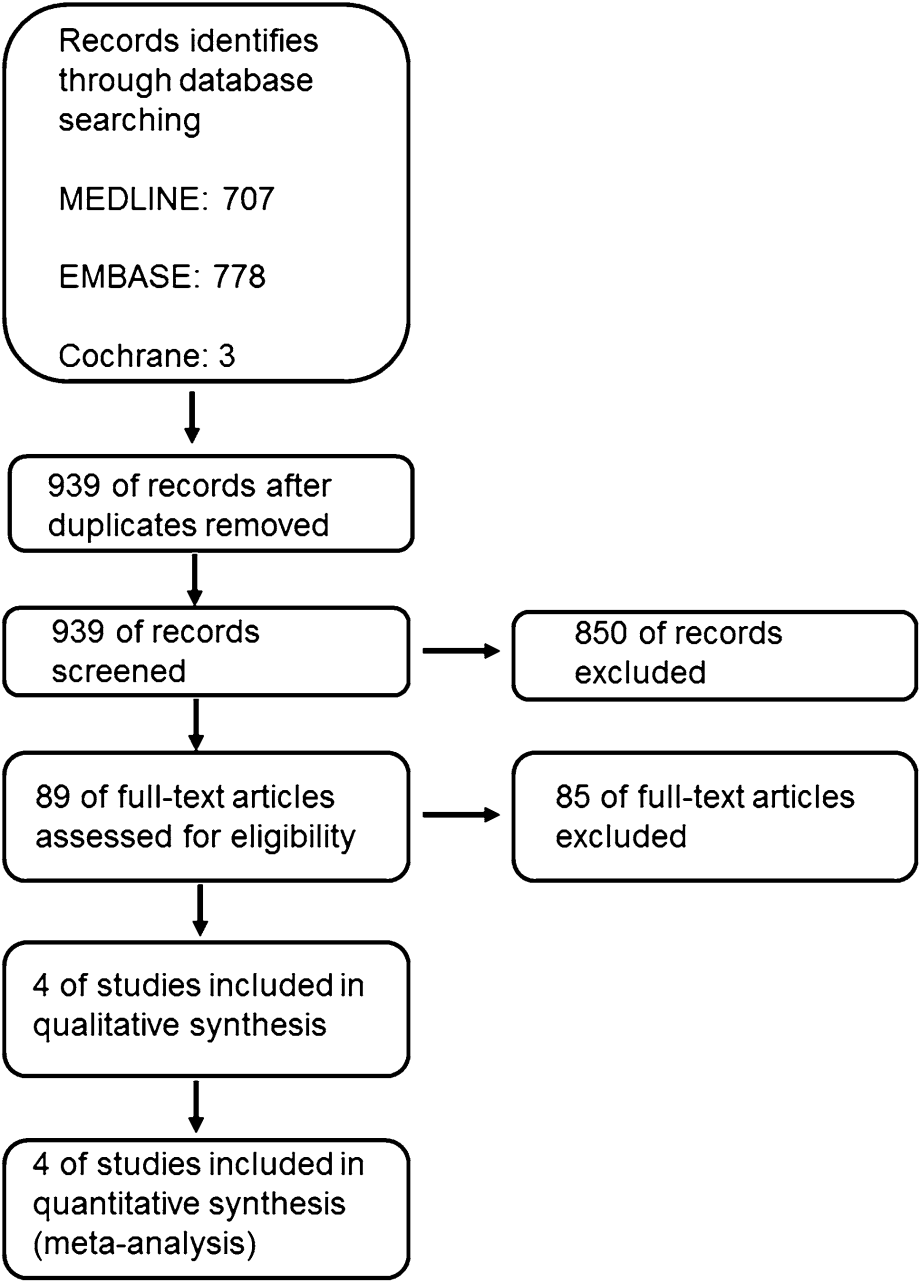
Flow diagram for data extraction according to PRISMA statement

We identified 939 articles after removing duplicates. 850 articles were excluded during title and abstract screen. Then, the full-text screen was performed and four retrospective cohort studies met the inclusion criteria (Fig. 1) [25–28]. No RCT was found, thus meta-analyses were performed only for observational studies. All four included studies reported the primary outcome postoperative esophageal stricture. A total of 362 patients were included for meta-analysis of postoperative stricture: 192 patients who received PARM and 170 controls. No study reported recurrent pneumonia, thus we did not perform meta-analysis for this secondary outcome.

Characteristics of included studies are shown in Table 1. Demographic data of one study was collected from their previous publication of the same group [29]. The confounders for each study are shown in Table 2. Of the four included studies, three were single center cohorts and the remaining was a multicenter cohort. In the multicenter cohort study, logistic regression analysis was used to evaluate esophageal stricture [25], and we were unable to extract data for each confounder in the patients with or without PARM. In three out of four studies, esophageal stricture was defined as symptomatic stricture which needed dilatation [26–28]. In the other study, esophageal stricture was diagnosed clinically by the responsible consultant surgeon [25]. Two of four studies included patients with EA type C [25, 26], whereas the other two studies included all types of EA [27, 28]. PARM consisted of PPI in two studies and H_2_ blocker in one study. In the remaining multicenter study, the type of PARM was variable with the majority of patients having H_2_ blockers (73%), and 16% having PPI. Duration of PARM administration varied among the studies and was not reported in one study. Follow-up periods varied between 1 and 5 years.

**Table 1:** Characteristics of included studies in the meta-analysis

Study	Study design	Country	Years of study	Sample size	PARM			Follow-up period	Reported outcome
					Type	Dose	Duration		
Allin et al. (2014) [25]	Retrospective cohortMulticenter	UK and Ireland	2008–2009	PARM 57 Control 19	H_2_ blocker 73% PPI 16%	NA	NA	1 year	Stricture diagnosed by consultant
Murase et al. (2015) [26]	Retrospective cohortSingle center	Japan	PARM 2010-2013 Control 2004–2009	PARM 13 Control 14	H_2_ blocker	1 mg/kg/day	At least 6 months	1 year	Stricture required dilatation
Stenstrom et al. (2017) [27]	Retrospective cohortSingle center	Sweden	PARM 2001-2014 Control 1983–1995	PARM 65 Control 66	PPI	2 mg/kg/day	3 months (2001–2009)12 months (2010–2014)	at least 1 year	Stricture required dilatation
Donoso (2016) [28]	Retrospective cohortSingle center	Sweden	PARM 2005-2013 Control 1994–2004	PARM 57 Control 71	PPI	1 mg/kg/day	Median 18 months	1 to 5 years	Stricture required dilatation

*PARM* prophylactic anti-reflux medicine, *PPI* proton pump inhibitor, *NA* not available

**Table 2:** Reported confounders in each study

Study		Long gap (%)	Type of EA(C/A/other)	Anastomotic leak (%)	Anastomotic tension (%)	Primary anastomosis (%)	Birthweight(< 1500 g/1500–2500 g/>2500 g)
Allin et al. (2014) [25]	PARM	NA	57/0/0	NA	NA	NA	NA
	Control	NA	19/0/0	NA	NA	NA	NA
Murase et al. (2015) [26]	PARM	7.7 (1/13)	13/0/0	7.7 (1/13)	NA	100 (13/13)	2/6/5
	Control	7.1 (1/14)	14/0/0	7.1 (1/14)	NA	100 (14/14)	0/8/6
Stenstrom et al. (2017) [27]	PARM	NA	63/2/0	10.8 (7/65)	NA	100 (65/65)	3/14/48*
	Control	NA	NA**	15.2 (10/66)	NA	100 (66/66)	5/26/35
Donoso (2016) [28]	PARM	14.0% (8/57)	45/5/7	7.0 (4/57)	33.3% (19/57)*	78.9% (45/57)	3/20/34
	Control	7.0% (5/71)	61/5/5	7.0 (5/71)	52.1% (37/71)	85.9% (61/71)	5/20/46

*EA* esophageal atresia, *PARM* prophylactic anti-reflux medicine, *NA* not available

*p < 0.05: PARM versus control, **reported as type C/type *A* = 13%/72%

**Fig. 2:**
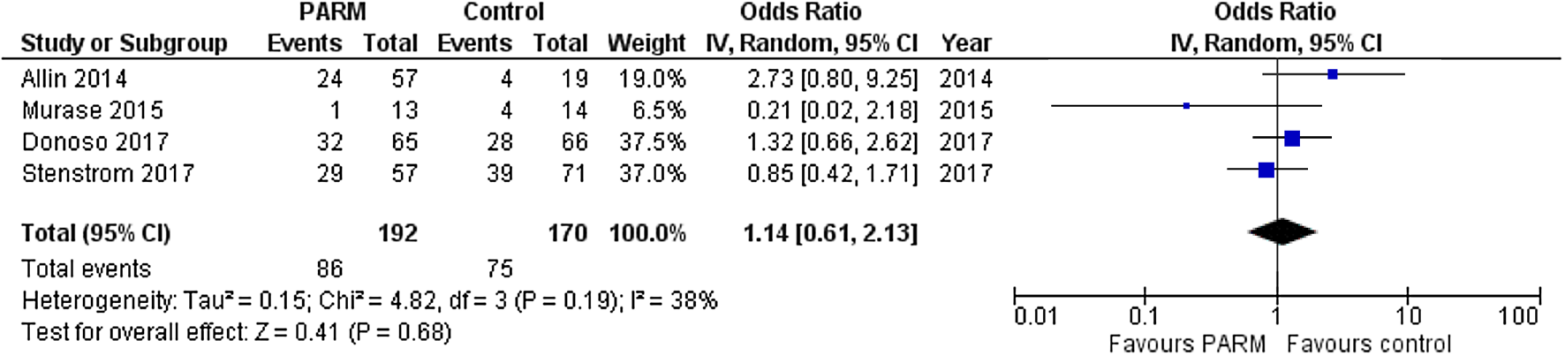
Forest plot of esophageal stricture after PARM versus control

Our meta-analysis showed that the incidence of esophageal stricture was 44.8% (86/192) in PARM group compared with 44.1% (75/170) in control group. There was no significant difference in the incidence of esophageal stricture between the two groups (OR: 1.14 95% CI: 0.61–2.13, *p* = 0.68, *I*^2^ = 38%) (Fig. 2).

Evidence for GRADE assessment is shown in Table 3. Outcomes from all four studies had low risk of bias according to NOS, as they were scored 7 or more (Table 4). Overall, we estimated the risk of bias in this systematic review as low. Inconsistency was not considered to be serious as heterogeneity was low (*I*^2^ = 38%). Indirectness was also considered not serious. Overall OIS was estimated as 926. Our result did not meet OIS, and imprecision was considered serious. As this meta-analysis included only four studies, we did not perform funnel plot analysis. There was no evidence to support publication bias. Because of imprecision, we rated down the quality of the evidence, and there was no reason for rating up the quality of the evidence. Overall, the quality of the evidence in this systematic review was assessed as “very low.”

**Table 3:** Evidence table for GRADE assessment

Quality assessment							No.of patients		Effect		Quality
№ of studies	Study design	Risk of bias	Inconsistency	Indirectness	Imprecision	Other considerations	PARM	Control	Relative	Absolute	
									(95% CI)	(95% CI)	
Esophageal stricture											
4	Observational studies	Not serious	Not serious	Not serious	Serious^a^	None	86/192 (44.8%)	75/170 (44.1%)	OR 1.14	33 More per 1000	⨁◯◯◯
									(0.61 to 2.13)	(From 116 fewer to 186 more)	Very low

*CI* confidence interval, *OR* odds ratio, *PARM* prophylactic anti-reflux medicine

^a^OIS was not met

**Table 4:** Newcastle–Ottawa Quality Assessment Scale for systematic review

Study	Selection				Comparability	Outcome				Score
	Representativeness of the exposed cohort	Selection of the non exposed cohort	Ascertainment of exposure	Demonstration that outcome of interest was not present at start of study	Comparability of cohorts on the basis of the design or analysis	Outcome	Assessment of outcome	Was follow-up long enough for outcomes to occur	Adequacy of follow-up of cohorts	
Allin et al. (2014) [25]	✦	✦	✦	✦	–	Stricture	✦	✦	✦	7
Murase et al. (2015) [26]	✦	✦	✦	✦	✦✦	Stricture	✦	✦	✦	9
Stenstrom et al. (2017) [27]	✦	✦	✦	✦	✦	Stricture	✦	✦	✦	8
Donoso (2016) [28]	✦	✦	✦	✦	✦✦	Stricture	✦	✦	–	8

One diamond symbol indicates one point

## Discussion

Recent surveys revealed the current trend of PARM use for patients after EA repair. Burge et al. reported that 51.6% of patients in the UK and Ireland are prescribed PARM, with the most common agents being H_2_ blockers [8]. Shawyer et al. reported that 84% of pediatric surgeons, mainly in Canada and the US, used PARM, with approximately equal proportions of PPI and H_2_ blockers [9]. In their report, patients were kept on PARM for variable lengths of time: 3 to 6 months (37%), or 6 to 12 months (35%). Lal et al. reported that 90% of US patients (data from the Midwest Pediatric Surgery Consortium) took PARM, most commonly PPI (40%), followed by H_2_ blockers (37%) [10]. These surveys revealed that the majority of patients are prescribed PARM after EA repair, with similar proportions of PPI and H_2_ blockers. In addition, recent guidelines published by ESPGHAN and NASPGHAN recommended that GER be treated with acid suppression in all EA patients in the neonatal period, as per expert opinion [30]. This guideline also recommended PPI as the first choice of PARM type. However, current evidence regarding the efficacy of PARM appeared insufficient. Thus, we conducted the present review to reveal the current evidence surrounding PARM use, with assessment of the quality of the evidence.

In the present review, we extracted 4 observational studies. Unfortunately, all 4 studies reported exclusively esophageal stricture as an outcome. This meta-analysis indicates that current evidence does not support the use of PARM to prevent esophageal stricture. However, we assessed quality of the evidence in the present review as “very low.” In consideration of the present results and the widespread use of PARM, as revealed by several surveys, well-controlled studies are needed to strengthen the quality of evidence for the need of PARM after EA repair.

Issues that need to be addressed to appropriately conduct future well-designed controlled studies are related to be the type, duration and dose of PARM. In this review, two studies used PPI and one used H_2_ blockers. Van Biervliet et al. reported that high doses of PPI were beneficial for patients with recurrent esophageal stricture which was resistant to H_2_ blocker [31]. Due to stronger acid-blocking effects, ESPGHAN and NASPGHAN guidelines recommend PPI as the first type of PARM to be used. However, as described previously, recent surveys revealed that PPI and H_2_ blockers are used in similar proportions. There seemed to be a lack of evidence regarding the most appropriate type of PARM. Duration of PARM administration is also controversial. There was great variability regarding duration of PARM among the included studies in this review. Stenstrom et al. reported that esophageal stricture after EA repair was not reduced by prolonged prophylactic PPI, comparing 12 months with 3 months [32]. On the other hand, ESPGHAN and NASPGHAN guidelines recommend that the duration of PARM administration should be one year or more, because complications due to GER can occur after 1 year of age, although they are more common within the first year of life. Safety and feasibility of PARM administration should also be taken into consideration. A recent systematic review about PPI for infants described a lack of evidence supporting the safety of PPI during infancy [33]. Brown et al. reported that children taking H_2_ blockers had a significantly higher risk of *Clostridium difficile* infection [34]. These side effects should be taken into consideration when selecting the most appropriate duration of PARM administration. In our study, there were also differences in duration of follow-up. Most patients were followed for at least 1 year, and, therefore, were not excluded from this meta-analysis.

To obtain more reliable results with higher quality of evidence, prospective studies are needed, which include well-controlled patient demographics and criteria for PARM use and follow-up. In future studies, analyses should also focus on safety and feasibility of PAR. In addition, other outcomes such as respiratory complications need to be evaluated.

## Conclusion

The present systematic review and meta-analysis indicate that the current literature does not support the use PARM to prevent the development of stricture after EA repair. However, the quality of the current evidence is very low. Thus, well-controlled prospective studies are needed.
